# Chromosome instability and expression of BRAF, TERT and P53 in macrophage murine cell line (J774-1)

**DOI:** 10.1186/1753-6561-8-S4-P47

**Published:** 2014-10-01

**Authors:** Lorena Polloni, Jessica Brito de Souza, Sofia Antunes Miranda, Pedro Henrique Alves Machado, Robson José Oliveira Júnior, Sandra Morelli

**Affiliations:** 1 Discente do Curso de Biotecnologia da Universidade Federal de Uberlândia, MG, Uberlândia, Brazil; 2 Discente do Curso de Ciências Biológicas da Universidade Federal de Uberlândia, MG, Uberlândia, Brazil; 3 Laboratório de Citogenética, Instituto de Genética e Bioquímica, Universidade Federal de Uberlândia, MG, Brazil

## Background

Cancer origin is closely linked with mutations and changes in the chromosome structure, making important such cytogenetics studies [1]. Macrophage murine cell line J774-1 is derived from a reticulum cell sarcoma and presents various characteristics of primary macrophages as synthesis and lysozyme secretion, phagocytosis, Fc receptor expression [2,3]. This work aimed to analyze the fundamental number and the constitutive heterochromatin using the staining with the flurochrome Hoechst 33258. In order to undertand possible changes in gene expression pattern, it was also quantified the levels of expression of the following important classes of genes: (A)-Genes responsible to promoting cell proliferation as BRAF and Telomerase (TERT); (B)-The P53 gene responsible by tumor suppressing.

## Methods

To obtain mitotic chromosomes, cells were grown in RPMI-1640 medium supplemented with 10% fetal bovine serum and antibiotics. Culture bottles were kept at 37 ° C in a CO2 incubator. After reaching approximately 80% confluence, cells were treated with 0.01% colchicine solution for three hours and trypsinized. The sample was treated with 0.075 M KCl hypotonic solution and fixed in Carnoy (methanol: acetic acid - 3:1 v/v). The cells were transferred to histology slides, stained with 5% Giemsa and examined under an optical microscope. To analyse constitutive heterochromatin, slides were stained with the A-T specific fluorochrome Hoechst 33258. For analysis of genic expression was performed real time PCR according to the manufacturer's instructions.

## Results and conclusions

The chromosome fundamental number of J774-1 cell line was very variable, ranging from 32 to 79, with the majority of metaphases with chromosome numbers concentrated between 72 and 73. This result indicates that J774-1 cell is a heterogeneous cell line. Hoechst staining showed that the constitutive heterochromatin of this cell line is A-T "rich" and it is concentrated in the pericentromeric region, corroborating with data observed in other murine cell lines and normal murine cells. Using real time PCR, it was detected a higher expression of BRAF and TERT genes in relation to control (whole blood cells) but comparing the expression of BRAF and TERT genes of J774-1 cells with other murine cell lines (S180, B16F10, MEF, NIH-3T3), the analyzed cell line showed lower levels of expression of these genes. In relation to P53 gene, it was detected higher expression levels in relation to control and all the other cell lines. High P53 expression was not expected in tumor or immortalized cells, once that its role is proliferation inhibition, but according to Campsi (2005) [4], even presenting intact function of tumor suppressors as P16 and P53 cells can acquire immortalized phenotype having an indefinite proliferation potential^4^. Cytogenetic results indicate high chromosome instability, with the presence of numerical and structural chromosome aberrations. The high fundamental number suggests a polyploidization event, probably due to endoreduplication, once many metaphases with diplochromosomes were found. Polyploidizadion may be the event that triggered genetic instability, generating chromosomal abnormalities and changes in gene expression, both events very important in the carcinogenesis.
